# Paradigms of Protist/Bacteria Symbioses Affecting Human Health: *Acanthamoeba species* and *Trichomonas vaginalis*

**DOI:** 10.3389/fmicb.2020.616213

**Published:** 2021-01-07

**Authors:** Fiona L. Henriquez, Ronnie Mooney, Timothy Bandel, Elisa Giammarini, Mohammed Zeroual, Pier Luigi Fiori, Valentina Margarita, Paola Rappelli, Daniele Dessì

**Affiliations:** ^1^School of Health and Life Sciences, University of West Scotland, Paisley, United Kingdom; ^2^Dipartimento di Scienze Biomediche, Università degli Studi di Sassari, Sassari, Italy; ^3^Mediterrenean Center for Disease Control, Sassari, Italy

**Keywords:** *Acanthamoeba*, microbial pathogenesis, mycoplasma, infection, *Trichomonas vaginalis*, microbial symbiosis

## Abstract

Ever since the publication of the seminal paper by Lynn Margulis in 1967 proposing the theory of the endosymbiotic origin of organelles, the study of the symbiotic relationships between unicellular eukaryotes and prokaryotes has received ever-growing attention by microbiologists and evolutionists alike. While the evolutionary significance of the endosymbiotic associations within protists has emerged and is intensively studied, the impact of these relationships on human health has been seldom taken into account. Microbial endosymbioses involving human eukaryotic pathogens are not common, and the sexually transmitted obligate parasite *Trichomonas vaginalis* and the free-living opportunistic pathogen *Acanthamoeba* represent two unique cases in this regard, to date. The reasons of this peculiarity for *T. vaginalis* and *Acanthamoeba* may be due to their lifestyles, characterized by bacteria-rich environments. However, this characteristic does not fully explain the reason why no bacterial endosymbiont has yet been detected in unicellular eukaryotic human pathogens other than in *T. vaginalis* and *Acanthamoeba*, albeit sparse and poorly investigated examples of morphological identification of bacteria-like microorganisms associated with *Giardia* and *Entamoeba* were reported in the past. In this review article we will present the body of experimental evidences revealing the profound effects of these examples of protist/bacteria symbiosis on the pathogenesis of the microbial species involved, and ultimately their impact on human health.

## Introduction

The relationships between protists and bacteria has been the subject of many evolutionary studies and there is a now a general understanding that protist evolution is highly dependent on the presence of bacteria. For example, horizontal gene transfer (HGT), the transfer of genetic material from bacteria to protist hosts, contributes considerably to protist genomes (López-García et al., 2017). Most of these studies have been addressed in environmental protists, where symbiosis between prokaryotic and protist host partners can be classified, based mainly on trophic interactions, as mutualistic, commensalistic or amensalistic. Endosymbiosis, the existence of one organism within another, is not uncommon between protists and bacteria. Indeed the evolution of these relationships has occurred separately across many lineages and plays a significant role in allowing adaptation of both species to new ecological niches (Nowack and Melkonian, 2010; Wernegreen, 2012).

However, less understood is the role these interactions play with regards to pathogenic protists. The exposure of these pathogens to potential endosymbionts varies dependent on the nature of the protist’s parasitism, be it obligate or opportunistic, or on the site of host infection. Many pathogenic protists infect apparent sterile environments (blood, central nervous system, and muscle tissue), but others are able to invade tissues in organs colonized by billions of microorganisms (e.g., intestinal tract, urogenital tract). The interaction of protists and bacteria in these environments is important to understand as it may influence pathogenesis and host immune responses. Obligate parasites are reliant on the host organism for survival, thus their exposure to potential endosymbionts is dependent on the niche they exploit within their host, while opportunistic parasitic protists are normally free-living in the environment, but can inadvertently infect a human host. In the environment, they cohabit ecological niches with many other microorganisms and are often able to predate and kill bacteria. However, in some cases, bacteria are able to resist intracellular killing and digestion, establishing endosymbiotic relationships and leading to profound changes to protist lifestyle. While the evolutionary significance of the endosymbiotic associations within protists has emerged and is intensively studied, the impact of symbiotic protist-bacteria relationships on human health has been seldom taken into account by researchers. Microbial endosymbioses involving human eukaryotic pathogens are not common, and the sexually transmitted *Trichomonas vaginalis* and the free-living *Acanthamoeba* species present two interesting cases in this regard.

In this review we will focus on the biology, epidemiology, pathogenesis of two paradigmatic examples of symbioses between protists and bacteria which have different lifestyles, opportunistic pathogen and obligate parasite, respectively and uniquely have impact on human health:

1. Multiple symbiotic *Acanthamoeba* species-bacteria relationships and the development of amoeba-resistant bacteria.
2. The first and unique case of two obligate human pathogens involved in a symbiotic relationship: *T. vaginalis* and the bacterium *Mycoplasma hominis.*

### Acanthamoeba

*Acanthamoeba* spp. are ubiquitous microorganisms, ordinarily free living within the environment. They have an active trophozoite form, approximately 15–35 μm in size with specialized pseudopodia and no descript shape, and a dormant cyst stage, approximately 10–20 μm in size, encased in a double-cellulose cell wall that provides increased protection from environmental stressors (Khan, 2006; Garajová et al., 2019). Globally, increasing cases of *Acanthamoeba*-associated infections have been documented, prompting significant concerns from a clinical perspective (Carnt et al., 2018; Randag et al., 2019; Scruggs et al., 2019; Höllhumer et al., 2020). These amoebae can be viewed as opportunistic parasites, ordinarily free living yet capable of causing severe infections under suitable circumstances. Infection of the eye, termed *Acanthamoeba* keratitis (AK), is predominantly associated with contact lens wear, while infections of the skin and central nervous system, cutaneous Acanthamoebiasis (CA) and granulomatous *Acanthamoeba* encephalitis (GAE), respectively, are linked with host immunodeficiency (Khan, 2015). Diagnosis of *Acanthamoeba* infections varies between diseases; in AK, direct microscopic observations, culturing techniques and molecular methods are used. For CA, biopsy staining techniques are useful and in GAE, diagnosis is often only possible after the death of the patient (Fung et al., 2008; Lorenzo-Morales et al., 2015). Similarly, treatment varies between pathologies, typically combined drug therapies are required with arduous treatment regimens and often surgical intervention is necessary to remove infected tissue. Drugs with significant anti-acanthamoebocide activity to date include anti-fungal medications such as ketoconazole and voriconazole, anti-protozoals such as pentimidine, aminoglycocides such as Paromomycin, sulfa drugs such as Sulfadiazine and alkylphosphocholines such as Miltefosine (Helton et al., 1993; Oliva et al., 1999; Tunkel et al., 2008; Dart et al., 2009; Lorenzo-Morales et al., 2015; Salameh et al., 2015; Kalra et al., 2020; Mooney and Williams, 2020). Several factors complicate the treatment and prevention of *Acanthamoeba* infections. For example, the ability to convert freely between a vegetative trophozoite stage to a dormant cyst stage or because of the metabolic similarities between *Acanthamoeba* cells and mammalian cells.

One issue that is often less considered is the connection between the environmental interactions of *Acanthamoeba* with bacterial symbionts and how this translates to synergistic pathogenicity against humans. Co-infections have been observed in all *Acanthamoeba* pathologies with multiple virus, bacteria and fungi species (Grayson, 2011; Pietrucha-Dilanchian et al., 2012; Scheid and Schwarzenberger, 2012; Guimaraes et al., 2016; Geith et al., 2018; Lau et al., 2019; Raghavan et al., 2019). However, the number of cases of CA and GAE is rare, with the latter proving fatal in ∼90% of cases (Król-Turmiñska and Olender, 2017). As such, the relevance of co-infection to clinical outcome is not assessed here, instead we focus on AK and bacterial co-infections. Interactions between *Acanthamoeba* and bacteria within the environment, how these interactions can exacerbate AK infections and some lesser considered issues that might arise from bacteria-protist relationships are discussed.

### *Acanthamoeba* Are Bacterivores

The nutritional requirements of *Acanthamoeba* are derived from their environment using both non-specific pinocytosis and receptor-mediated phagocytosis (Chambers and Thompson, 1976; Alsam et al., 2005). Phagocytosis allows for the active predation and digestion of bacteria and grazing of free-living amoebae has a significant role in maintaining healthy ecosystems. It is estimated that free-living amoebae consume up to 900 g of bacteria per meter square annually, aiding in the regulation of bacterial communities by as much as 60% (Clarholm, 1981). While important ecologically, the microbial interactions of *Acanthamoeba* with bacteria can have substantial clinical implications. The process of phagocytosis in *Acanthamoeba* is not fully understood, however the organism shares remarkable similarities with human immune cells such as macrophages (Siddiqui and Khan, 2012). The known processes in *Acanthamoeba* include an initial adhesion mediated by mannose-binding protein (MBP) and an actin dependent process involving polymerization of monomeric G-actin into filamentous F-actin (Alsam et al., 2005). The engulfed particles are stored within phagosomes, these phagosomes then fuse with cytoplasmic vacuoles termed lysosomes to form phagolysosomes. Lysosomes provide the necessary enzymes and free radicals needed to degrade organic material for use as a food source (Allen and Dawidowicz, 1990; Hong et al., 2018). The microorganism(s) within *Acanthamoeba* phagolysosomes are degraded by oxidative stress, antimicrobial products, and acidification (Haas, 2007; German et al., 2013). It appears these phagosomes can sense whether these products are degradable, with non-organic beads distinguished from degradable organic matter in *Acanthamoeba* (Bowers and Olszewski, 1983). It is well understood that bacteria can evade predatory cells by either preventing detection or uptake as a result of poor receptor binding (Uribe-Quero and Rosales, 2017). This has been primarily observed in the mammalian immune response to bacteria (Uribe-Quero and Rosales, 2017), but holds relevance to the free-living amoebae, such as *Acanthamoeba*, due to their resemblance of human immune cells (Siddiqui and Khan, 2012). The selectivity demonstrated by Bowers and Olszewski (1983) however is pertinent in that there are multiple reports of phagocytosed bacteria able to survive cell digestion, giving rise to a situation of endosymbiosis and complicating *Acanthamoeba* related infections by introducing co-infections and delaying accurate diagnosis (Schmitz-Esser et al., 2008).

### Amoeba-Resistant Bacteria (Phagocytosis Resistance)

**FIGURE 1:**
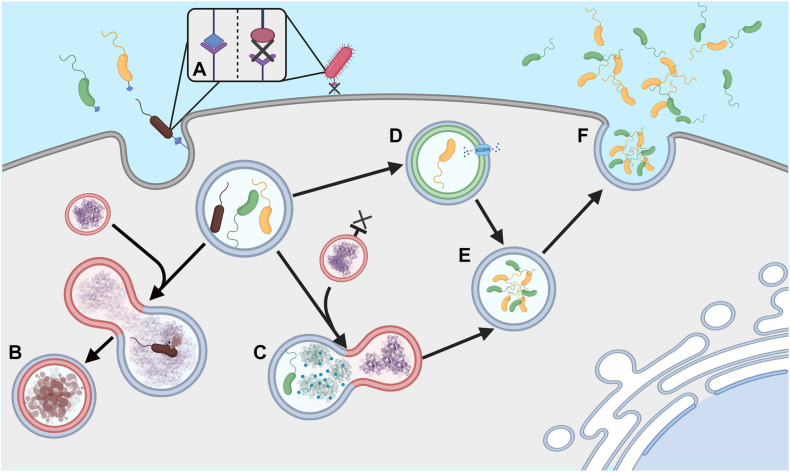
Phagocytosis evasion strategies **(A)** Ingestion of extracellular bacteria is triggered by complementary membrane bound receptors; absence of these receptors can allow evasion of phagocytosis. **(B)** Ordinarily phagosomes fuse with lysosomes to form phagolysosomes and degrade engulfed bacteria. **(C)** Some bacteria can neutralize lysosome attack, **(D)** others can prevent lysosome formation and form microenvironments, allowing nutrients to be obtained from the host *Acanthamoeba*. **(E)** Survival within these macrophages can facilitate cell division in some species, **(F)**. Cells can then be released into the extracellular environment to re-infect other amoebae, often leading to death of the host amoeba. Created with BioRender.com.

*Acanthamoeba* have been shown to harbor a large number of viral, bacterial and fungal species, although not always to their own benefit. For example, the bacterium *Legionella pneumophila* can be detrimental to *Acanthamoeba*, surviving and replicating within the host and killing the amoeba upon escape (Rowbotham, 1980). In other instances, the inter-organism relationship can prove mutually beneficial with several endosymbionts existing within the cytoplasm of the amoeba (e.g *Alphaproteobacteria* spp*., Chlamydia* spp. and *Bacteroidetes* spp.) (Schmitz-Esser et al., 2008). The mechanisms in which bacteria use to allow survival within free-living amoebae vary between species and this variance coupled with the phylogenetic differences between the bacteria suggests the ability has occurred multiple times over the organism’s evolutionary history (Schmitz-Esser et al., 2008). Avoiding phagocytosis is complex and the strategy used differs between organisms (Figure 1). For example, *L. pneumophila* is capable of forming a membrane-bound microenvironment, accessing nutrients through membrane transporters, endoplasmic reticulum manipulation and fusion with other cytoplasmic vesicles (Hervet et al., 2011; Harding et al., 2013; Michard et al., 2015). In contrast, it has been suggested that *Vibrio cholerae* can resist degradation using a complex neutralizing strategy, preventing the pH, reactive oxygen and nitrogen species, digestive enzymatic activity, and antimicrobial peptide products from exerting their toxicity (Espinoza-Vergara et al., 2020). Other strategies can be inferred from data gathered in macrophage work, for example receptor avoidance strategies that prevent ingestion, or interference with the formation of phagolysosomes within the cell (Flannagan et al., 2015; Uribe-Quero and Rosales, 2017).

**FIGURE 2:**
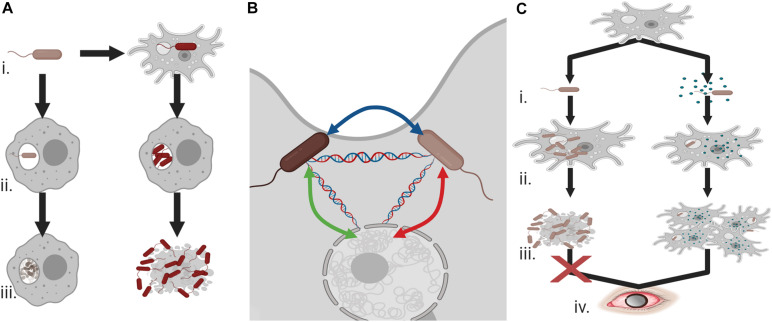
Endosymbionts and pathogenicity **(A) (i)** Co-existence of bacteria and *Acanthamoeba* can allow for the evolution of phagocytic resistant cells, **(ii)** unlike bacteria with no exposure to the amoeba these *Acanthamoeba*-associated bacteria can resist macrophage attack and undergo cell division, and **(iii)** rather than being degraded, they can cause cell death and demonstrate increased cytotoxicity against human cells. **(B)** *Acanthamoeba* can provide an ideal environment for the transfer of antimicrobial resistance or virulence genes between endosymbiotic bacteria and the protist. **(C) (i)** *Acanthamoeba* and *Legionella pneumophila* interactions can be balanced by the presence of an additional endosymbiont, **(ii)** intracellular division of *L. pneumophila* is prevented in the presence of these endosymbionts, **(iii)** allowing host survival, and **(iv)** ultimately increasing the potential for *Acanthamoeba* keratitis infections. Created with BioRender.com.

In addition to survival within phagosomes, other bacterial species are capable of survival within the cytoplasm of the amoebae and unlike the aforementioned organisms are in fact predominantly beneficial in nature. The web of interactions is complex with regards to the bacteria–protist relationship and how this impacts pathogenicity (Figure 2). Recent work has documented an increased ability to uptake pathogenic bacteria when *Acanthamoeba* also possess specific endosymbionts (Okubo et al., 2018), the advantage of the endosymbiont however is in that it appears to prevent cell division from the engulfed bacteria. For example, intracellular cell division of *L. pneumophila* is inhibited in the presence of *Neochlamydia* within the host *Acanthamoeba* (Okubo et al., 2018). Survival within the amoebae is beneficial to the bacteria, not only because it prevents predation but because it offers opportunity to alleviate extracellular pressure, particularly given the cyst forming ability of the protist. HGT is also observed as a result of these relationships between both amoebae and bacteria and between the various intracellular bacteria within the protist (Wang and Wu, 2017) that has the potential to contribute to the emergence of drug resistant organisms.

### *Acanthamoeba* Keratitis

The most well-researched and common pathology associated with *Acanthamoeba* is AK, a sight threatening condition resulting from infection of the eye. In developed countries, AK is largely associated with poor contact lens hygiene, with 95% of AK cases linked with contact lens wearers (Stapleton and Carnt, 2012). Micro-lesions in the cornea, such as those caused by contact lens wear, provide an entry point for the amoebae (Marciano-Cabral and Cabral, 2003), interactions with the Bowman’s membrane and stroma on the cornea of the eye using proteins such as mannose or laminin binding proteins then allow for adhesion and infection (Panjwani and Yang, 1997; Hong et al., 2004; Ng et al., 2017). *In vitro*, studies have shown that *Acanthamoeba* trophozoites can kill corneal epithelial cells, corneal endothelial cells, corneal fibroblasts, iris ciliary body cells via cytopathic processes such as cytolysis, phagocytosis, and apoptosis (Khan, 2001; Hurt et al., 2003; Clarke and Niederkorn, 2006).

Generally, the symptoms of AK infections can include redness of the eye, blurred vision, sensitivity to light, excessive tearing, ring infiltrate, and severe eye pain (Niederkorn et al., 1999). A more comprehensive timeline of AK infection however has been detailed by Szentmáry et al. (2019), in which the clinical presentation of symptoms is as follows; chameleon-like epithelial changes and multifocal stromal infiltrates occur within the first two weeks followed by a ring infiltrate or Wesseley immune ring and perineural infiltrate in the first month of infection and in late infections sterile anterior uveitis, scleritis, broad-based anterior synechiae, secondary glaucoma, iris atrophy, mature cataract, chorioretinitis, and retinal vasculitis have all been observed (Szentmáry et al., 2019). The inflammation induced by the reaction of the eye to the protist can eventually cause blindness (Lorenzo-Morales et al., 2013). Comprehensive reviews have been published recently on AK (Maycock and Jayaswal, 2016; Garg et al., 2017; Szentmáry et al., 2019), however, the role of secondary infections in the disease is less considered. Though *Acanthamoeba* is the dominant organism that should be treated in AK, co-infection is not uncommon and can oftentimes result in late diagnosis, a significant factor in disease outcome as will be discussed.

### Complex Microbial Acanthamoeba Keratitis

Complex microbial *Acanthamoeba* keratitis (CMAK) is the term used to describe AK involving secondary infectious agents such as infectious bacteria. Table 1 provides a summary of specific bacterial co-infections identified alongside AK to date. *Acanthamoeba* often act as a “Trojan horse” for these infectious bacteria by acting as a vector, essentially providing transport and opportunity to colonize the corneal surface of a susceptible host (Barker and Brown, 1994). Indeed, development of AK in rabbit corneas was shown to be reliant on the presence of the bacteria *Pseudomonas aeruginosa*, with bacterial density having an impact on development and severity of the infection while those inoculated in the absence of bacteria were unable to cause infection (Nakagawa et al., 2017). It is tempting to speculate from this study that the risk of AK in non-contact lens wearers is increased by the presence of a co-infectious agent and indeed this is a point that has been supported in recent investigations by Raghavan et al. (2019) in which co-infections were found to be less restricted to contact lens use than what is observed in *Acanthamoeba* infections alone (Stapleton and Carnt, 2012; Nakagawa et al., 2017; Raghavan et al., 2019). Other research has found bacterial infection in AK can enhance binding of both *Acanthamoeba* and bacteria to the corneal endothelial cells (Clarke and Niederkorn, 2006). The increased risk of infection supports further the need for compounds with a broad antimicrobial activity. Antimicrobial agents such as hydrogen peroxide (H_2_O_2_), quaternary ammonium compounds (QACs) or alkylphosphocholines (APCs) that are in use or under investigation at present and can be incorporated into disinfectants or contact lens solutions for prevention (H_2_O_2_ and QACs) or as part of the current AK treatment regimen (APCs) offer hope in this regard given their ability to target both *Acanthamoeba* life stages and the other co-infectious and endosymbiotic agents (Walochnik et al., 2002; Hiti et al., 2005; Carmona-Ribeiro and de Melo Carrasco, 2013; Wessels and Ingmer, 2013; Fears et al., 2018; Mooney et al., 2020; Wekerle et al., 2020).

**TABLE 1:** Detail on mixed infection of *Acanthamoeba* and bacterial keratitis in the literature.

Organism	Number of cases	% to total cases of bacterial keratitis
**Gram negative**		
*Enterobacteriaceae*	38	25.50
*Pseudomonas aeruginosa*	21	14.10
*Acinetobacter sp.*	5	3.35
*Stenotrophomonas maltophilia*	5	3.35
**Gram positive**		
*Staphylococcus aureus*	18	12.08
*Staphylococcus epidermis*	5	3.35
*Streptococcus pneumonia*	13	8.72
*α-haemolytic streptococci*	6	4.03
*Corynebacterium sp.^*a*^*	10	6.72
*Co-Staphylococcus*	23	15.45
*Serratia sp.^*b*^*	3	2.01
*Streptococcus viridians*	1	0.67
*Streptococcus oralis*	1	0.67
Total	**149**	**100%**

*^*a*^*Corynebacterium xerosis* was identified in two cases. ^*b*^*Serratia marcescens* was identified in two cases.*

The degree in which bacterial endosymbionts actively effect pathogenesis of AK presents contrasting results. It has been demonstrated that endosymbiont bacteria such as *Pseudomonas* and *Mycobacterium* can enhance corneal toxicity in patients with AK (Iovieno et al., 2010). The degree increase in corneal toxicity also varies depending on the endosymbiont species, *Pseudomonas* and *Mycobacterium* have shown enhanced corneal toxicity relative to *Legionella* (Iovieno et al., 2010). However, it is unclear whether this additional toxicity is caused by *Acanthamoeba* or its endosymbionts. More recently however, a retrospective analysis of AK and *Acanthamoeba*/bacterial keratitis revealed no significant differences in the disease presentation or the overall outcome to patients, perhaps testament to the broad antimicrobials used in current AK treatment regimens (Singh et al., 2020). Despite this, it is important to consider the increased difficulty of timely and accurate diagnosis during co-infections. AK data has long been considered underreported, the reasoning for this is the ease in which misdiagnosis or partial diagnosis can occur given the similarities to other forms of keratitis. Raghavan et al. (2019) note that in cases of *Acanthamoeba* co-infections, the need for corneal scrapings and diagnostic cultures were higher than those of AK alone (Raghavan et al., 2019), a factor that could impact treatment success given the rapid encystation *Acanthamoeba* can undergo when incorrect drug therapies are used. It is clear that more work is required to provide an accurate assessment of the direct risks of co-infection on patient outcome. The risks of delayed and mistaken diagnosis coupled with the increased ability of *Acanthamoeba* to infect human hosts in the presence of the bacterial symbionts should also be taken into consideration in this regard.

### The Trojan Horse of the Microbial World: An Evolutionary View

It is estimated that 22% of all *Acanthamoeba* isolates contain an additional pathogenic species (Guimaraes et al., 2016) and presumably a significant number of non-pathogenic species. For this reason, *Acanthamoeba* have been referred to as the “Trojan horse” of the microbial world (Barker and Brown, 1994). A factor less explored in the pathology of both the *Acanthamoeba* and bacterial infections, be it individually or as co-infections, are the ongoing environmental factors that contribute to the pathogens ability to infect the human host. As suggested previously, *Acanthamoeba* spp. can act as the host organisms for several *Legionella* spp. and recent work has indicated the implications these interactions can have on human health. Gomes et al. (2018) describe an increase in the expression of several virulence factors associated with macrophage pathogenicity when the bacteria are first incubated within the *Acanthamoeba* relative to those that had no prior host (Gomes et al., 2018). The same research notes that the expression levels of enzymes involved in type IV secretion were upregulated to varying degrees between two *Legionella* spp., shown previously to be involved in macrophage apoptosis in humans (Zink et al., 2002). *Acanthamoeba* derived species were also likely to elicit a more extreme immune response than their counterparts not exposed to the bacterial symbiont (Gomes et al., 2018). This finding gives support to the “training ground” hypothesis, eloquently described by Molmeret et al. (2005). Briefly, the close association between free-living amoebae and bacteria aids in the selection of bacteria resistant to the phagocytic behaviors of the protist, the continuous evolution of the phagocyte resistance strategies serve to increase pathogenesis of the organisms upon entry into the human host, capable of resisting macrophage attack (Molmeret et al., 2005).

While the evolution of mechanisms that prevent phagocytosis and the co-evolution of amoebic countermeasures is ongoing in the environment, there is a risk that the limited exposure in the human phagocytic counterparts could leave them significantly behind in the evolutionary arms race. Understanding these interactions could potentially open up new mechanisms of combatting phagocyte resistant bacteria in human infections. One such method already discussed is the presence of non-pathogenic endosymbionts in *Acanthamoeba. Neochlamydiae* for example provide protection against the pathogenic *Legionella* spp., preventing cell division within the phagosomes and interfering with the transition to the transmissible stage of the pathogen (Okubo et al., 2018; König et al., 2019). This same three-way interaction might have clinical implications however, the *Neochlamydia*-*Acanthamoeba* strains were capable of “backpacking” several strains of infectious bacteria; *E. coli, Salmonella* spp., *P. aeruginosa*, and *S. maltophilia* (Okubo et al., 2018). While the impact of this is unexplored at present, it could be speculated that the ability of endosymbionts to prevent cell death arising from other phagocyte-resistant bacteria while not impacting their overall viability might serve to introduce increased risks of co-infection into the human host. This co-existence also presents additional problems with multiple studies demonstrating a high degree of genetic exchange within the amoebae, HGT occasionally occurring between the amoebae and bacteria but more commonly between different intra-amoebal bacteria (Wang and Wu, 2017), providing an intracellular environment that could easily breed drug resistance, particularly when exposed to sub-lethal concentrations within the amoebae relative to the extracellular environment. Increased understanding of these interactions in the environment and *in-vivo* is important and might aid in the formulation of better diagnostic and treatment regimens.

### *Acanthamoeba* and Waterborne Bacteria: Resistance and Resuscitation

Free-living amoebae have the potential to act as a vector of transmission for several pathogenic waterborne bacteria. For example, the free-living amoeba *Willaertia magna* can excrete vesicles containing *L. pneumophila* cells in quantities sufficient to initiate human infections (Shaheen and Ashbolt, 2018), other free-living amoeba have been shown to protect *Helicobacter pylori* from disinfection in human food sources (Moreno-Mesonero et al., 2020) and several *Acanthamoeba* spp. have been found to harbor *L. pneumophila, Aeromonas* spp., and *Pseudomonas* spp. (Steinert et al., 1997; Rahman et al., 2008; Dey et al., 2019). The co-existence of these organisms within human-made water systems can have a significant impact on human health. *L. pneumophila* is the causative agent of Legionnaires’ disease, a disease which causes severe pneumonia and can result in death, *Aeromonas spp.* are associated with gastroenteritis, wound infections and bacteremia, *H. pylori* can cause severe abdominal issues and *Pseudomonas spp.* can cause several severe infections. While disinfection strategies are in place to prevent infection from these organisms, their efficacy is oftentimes hampered by the protist-bacteria relationship. This relationship not only facilitates drug resistance but reduces the efficacy of commonly used disinfection methods (e.g., chlorine, heat) in artificial water systems. The susceptibility of *L. pneumophila* to sodium hypochlorite for example is reduced 4-fold in bacteria that reside within *A. polyphaga* relative to exposed bacteria (García et al., 2007), unsurprising given the higher tolerance of *Acanthamoeba* to chlorine than *L. pneumophila* (Martin et al., 2020). Improved heat resistance in *L. pneumophila* at temperatures as high as 90^*o*^C has also been reported when co-cultured with *A. mauritaniensis* relative to bacteria monocultures (Dobrowsky et al., 2017). Thus, the increased survivability afforded by amoebae to these disinfection strategies should be taken into consideration to avoid the spread of bacterial pathogens in human-made water supplies.

Bacteria are not wholly reliant on amoebae for protection against environmental stressors however, by shifting to a viable but non-culturable (VBNC) form they can also decrease disinfection efficacy (Li et al., 2014). Existence within the VBNC form hampers standard methods of detection, indeed a recent study conducted by Casini and colleagues noted that while 87.2% of samples were deemed negative for *L. pneumophila* using culturing techniques, only 34.5% returned negative results after screening with real-time PCR (Casini et al., 2018). These VBNC bacteria might pose a significant risk to human health given the ineffectiveness of standard culture methods and as such new strategies for accurate detection have been proposed, imaging flow cytometry for example (Dey et al., 2019). Most pertinent to this review however is how amoebae-bacteria interactions influence this shift to and from the VBNC form. In *Aeromonas hydrophila*, the shift to the VBNC form occurs on average twenty days earlier in the presence of *Acanthamoeba castellanii* than it does alone (Rahman et al., 2008), reducing the window for its detection using standard culture techniques and increasing the likelihood of underreporting its presence in water systems and potentially the chances of human infection. Interestingly, amoebae are also involved in VBNC resuscitation; the shift back to the culturable, active and most importantly virulent form of the bacterium. The resuscitation of several species of VBNC bacteria upon being internalized by the amoebae has been documented (Steinert et al., 1997; García et al., 2007; Epalle et al., 2014; Casini et al., 2018; Dey et al., 2019, 2020), whereby they begin multiplying within the intracellular space of the amoeba. The exact reason for this shift is unknown and relatively unexplored, however it has been reported than the presence of extracellular pyruvate and glutamate can drive the shift to the replicative form, perhaps acting as antioxidants and facilitating cell recovery (Ducret et al., 2014). In understanding the driving forces behind these interactions it could allow for more effective culture methods to be developed that might increase detection of these pathogens.

The dangers of these interactions are significant, for example, the resuscitation of *L. pneumophila* in *Acanthamoeba* spp. is possible even after heat shock or chlorine treatments and upon shifting from the VBNC form the bacteria become fully virulent, capable of causing infections in mammalian cells and posing a major risk to human health (Steinert et al., 1997; García et al., 2007; Epalle et al., 2014). In *P. aeruginosa* this shift to the active form can occur within two hours of *Acanthamoeba* uptake (Dey et al., 2019), thus relatively small windows are required, emphasizing the need for maintained and uninterrupted disinfection strategies in human-made water systems. The aforementioned study by Casini et al. (2018) emphasizes this point further with the continuous use of low monochloramine concentrations for the disinfection of hospital water networks serving only to maintain *L. pneumophila* in the VBNC form that, on the removal of this stressor and exposure to *A. castellanii*, could re-enter the active form. While most studies investigate the interactions of *Acanthamoeba* with bacteria, other free-living amoebae should also be considered. Recently, Dey and colleagues demonstrated the potential for both *W. magna* and *Vermamoeba vermiformis* to not only carry and allow multiplication of *H. pylori* but to resuscitate the bacteria to a culturable state (Dey et al., 2020). It is becoming increasingly more evident that to minimize the risks of these pathogens to human health the whole microbial community should be considered, and further research should aim to better understand the interactions between these organisms.

## *Trichomonas vaginalis* and *Mycoplasma hominis*: Biology and Impact on Human Health of Two Sexually Transmitted Pathogens

### Trichomonas vaginalis

*Trichomonas vaginalis* is a flagellated protist which parasitizes the human urogenital tract causing trichomoniasis, the most common non-viral sexually transmitted disease in humans, with an estimated incidence of 156 million new cases per year worldwide (Rowley et al., 2019).

*Trichomonas vaginalis* is an obligate parasite unable to survive in the environment, and humans are the only known natural host. This protist does not have a cystic stage and exists in the typically pear-shaped trophozoite form only, which measures on average 10 μm × 7 μm. It has four anterior flagella and a fifth recurrent flagellum, incorporated in the free margin of an undulating membrane. *T. vaginalis* is characterized by the absence of mitochondria and by the presence of anaerobic hydrogenosomes, spherical organelles measuring from 200 nm to 1 μm involved in the parasite’s metabolic pathways (Fiori et al., 2016).

The infection in women is characterized by a severe vaginitis accompanied by abundant malodorous vaginal discharge, dysuria, itching, vulvar irritation, and abdominal pain. The infection tends to become chronic, particularly in women, and may persist for long periods. Interestingly, about 50% of women and up to 75% of men are asymptomatic.

Trichomoniasis is associated with severe complications, such as increased risk of HIV acquisition and of cervical and prostate cancer (Stark et al., 2009). The infection can also lead to adverse pregnancy outcomes, such as preterm delivery, premature rupture of membranes, and low birth weight (Margarita et al., 2020).

The standard treatment for trichomoniasis is a single 2-g dose of metronidazole or tinidazole. Nitroimidazole derivatives remain the sole treatment of trichomoniasis, and despite an ever-increasing number of metronidazole-resistant isolates having been reported in recent years, effective alternative therapies are not yet available.

Diagnosis of trichomoniasis has traditionally been based on direct microscopic examination of wet mount preparations, and it is still largely used in disadvantaged settings such as developing countries, due to its rapidity and low cost. However, its poor sensitivity (44–68%) is often compensated by culture-based diagnostic systems, with a sensitivity ranging from 81 to 94% (Schwebke and Burgess, 2004; Van Gerwen and Muzny, 2019). The reliability of these diagnostic tests is strongly dependent on the viability and on the number of trichomonads. Moreover, culture requires up to seven days of incubation and is therefore not widely used. New diagnostic methods that do not depend on the viability of the protist, such as nucleic acid amplification tests (NAATs), are now available and are widely used in laboratories, largely replacing traditional diagnostic approaches (Hobbs and Seña, 2013). They have the best sensitivity and specificity and are usually commercialized as multiplex detection assays in association with other sexually transmitted microorganisms, proving to be particularly suitable for testing asymptomatic patients, which are typically characterized by a low parasite load. Recently, a rapid immunochromatographic capillary-flow enzyme immunoassay, that has been commercialized as OSOM *Trichomonas* test, showed good performances with a sensitivity similar to that of NAATs; it can be used at the point of care and results are available within 15 minutes (Campbell et al., 2008).

The pathogenetic mechanisms of trichomoniasis have been the subject of intensive research over the years, and the current model involves several aspects of the host–parasite relationship, ranging from host epithelial cells damage and disruption of resident microbiota, to subversion of immune response and induction of local inflammation. Being an extracellular organism, the parasite must adhere to host epithelial cells to establish infection. To enhance the adhering surface, it changes its morphology from pear-shaped to amoeboid, generating a wider contact surface with vaginal epithelial cells (VECs). The protist is able to attach to a broad range of cells (Addis et al., 2000) and to adapt to the highly dynamic vaginal microenvironment. A high number of adhesion factors are involved in the first phase of infection (Hirt et al., 2012), which is followed by the production of several toxic products. The secretion of molecules with cytotoxic activity such as proteases and pore-forming proteins plays an important role in the epithelial cell damage observed during infection (Diaz et al., 2020).

Concurrently, the host response plays an important role in *T. vaginalis* pathogenesis, contributing with a marked local inflammation characterized mainly by a heavy neutrophil infiltration at the site of infection. Indeed, the host tissue damage appears to be an outcome of the concomitant action of parasite cytotoxins and of the inflammatory response (Mercer and Johnson, 2018).

### *Trichomonas vaginalis* and *Mycoplasma hominis*: The Discovery of a Symbiotic Relationship

In 1997 Koch et al. (1997) reported a clinical association between *T. vaginalis* and the bacterium *Mycoplasma hominis*. The following year, the observation that the large majority of *T. vaginalis* clinical isolates collected from a patient population of different geographic origin were stably infected by *M. hominis*, paved the way for the discovery of a symbiotic relationship between the two microorganisms (Rappelli et al., 1998), which is the first reported involving two obligate human parasites, capable to cause distinct diseases in the same organs. A subsequent number of papers investigated the association rate between the two symbionts in clinical isolates, showing a high degree of variability (5–89%) depending on the geographical setting of the studies (Fichorova et al., 2017).

*Mycoplasma hominis* is an obligate human bacterial parasite belonging to the class of *Mollicutes*, which comprises microorganisms with a small genome size and lacking a rigid peptidoglycan cell wall and many biosynthetic pathways (Taylor-Robinson, 2017). Altogether these features make the bacterium strongly dependent on the host, which provides a protected niche and nutrients necessary for survival. *M. hominis* colonizes the human urogenital tract, and its prevalence shows a high degree of variability, ranging from 1.3 to 51% (Diaz et al., 2010; Rumyantseva et al., 2019), depending on the population subject of the studies. Although the infection is in most cases asymptomatic, it is associated not only with alterations of the vaginal microbiota and bacterial vaginosis, but also with extragenital conditions. Moreover, *M. hominis* infection in pregnancy can cause severe complications, such as preterm birth and chorioamnionitis (Margarita et al., 2020). The virulence mechanisms exerted by the bacterium in these pathological conditions are still unclear. The descriptions and characterizations of mycoplasmal proteins involved in pathogenicity are sparse: OppA, P80, and P50/vaa are thought to play a role as adhesins (Kitzerow et al., 1999; Hopfe et al., 2004). Notably, several studies showed a marked proinflammatory host response to *M. hominis* surface lipoproteins via interaction with Toll-like Receptor-2 (TLR-2), leading to IL-23 production by dendritic cells (Goret et al., 2017). Furthermore, a predicted surface lipoprotein (MHO_0730) was shown to have a role in the interaction of *M. hominis* with neutrophils. MHO_0730 demonstrated a nuclease activity thought to contribute to the evasion of neutrophil response by disrupting the DNA backbone of Neutrophil Extracellular Traps (Cacciotto et al., 2019).

### *T. vaginalis/M.hominis* Symbiosis and Immunopathogenesis

**FIGURE 3:**
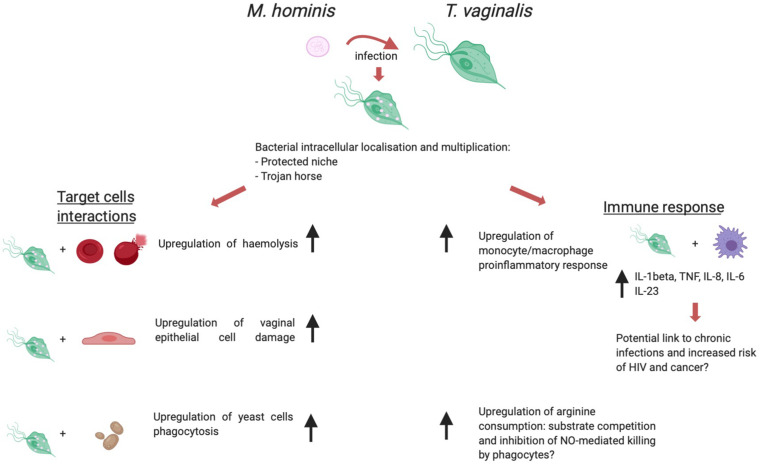
*T. vaginalis/M. hominis* symbiosis, a schematic view of the influence of the presence of *M. hominis* within trichomonad cells over immunopathogenesis. Created with BioRender.com.

The biological association between *T. vaginalis* and *M. hominis* has been shown to have a relevant impact on several aspects of the protist immunopathogenesis (summarized in Figure 3).

The first studies aimed at characterizing this symbiosis showed that *M. hominis* is able to localize and multiply in the intracellular compartment of *T. vaginalis*, although the specific subcellular localization (cytoplasmic vs inside vacuoles) was not assessed (Dessi et al., 2005). This localization may provide protection from antibiotics and host immune response to the bacterium. Additionally, Thu and colleagues (Thi Trung Thu et al., 2018) investigated the hypothesis of a potential mechanism by which *T. vaginalis* may function as a protected niche to transport virulent *M. hominis* to human cell cultures. Metronidazole treatment of mycoplasma-infected trichomonad cells led to cell stress and death, causing the release of viable bacteria which in turn actively infected cultured human cells, thus mimicking a potential situation which might occur *in vivo*. Together with the ability of naturally mycoplasma*-*infected *T. vaginalis* to transmit the mycoplasmal infection to a human-derived cell line and to naturally *M. hominis*-free trichomonad isolates (Rappelli et al., 2001), this suggests a role for *T. vaginalis* as a “Trojan horse” for *M. hominis*.

As previously discussed in this review article, the current model of *T. vaginalis* infection pathogenesis involves on the one hand the cytopathic effect directly induced by *Trichomonas* cells through adhesion to host cells and secretion of cytotoxins, and on the other hand the local inflammation evoked by the infection, both contributing to mucosal tissue damage (Mercer and Johnson, 2018). The symbiosis with *M. hominis* appears to affect both aspects of *T. vaginalis* immunopathogenesis. An *in vitro* model of interaction between *T. vaginalis* and VECs showed that *mycoplasma-*infected parasites can induce a more pronounced cellular damage to VECs when compared to *mycoplasma*-free control trichomonads (Vancini et al., 2008). This effect can be interpreted as tissue- and species-specific, since a similar upregulation of cytopathogenicity was not observed when the target cells were represented by the canine kidney cell line MDCK. Vancini and colleagues described in the same study that *M. hominis*-infected *T. vaginalis* displayed a marked increase in the phagocytic activity towards *Saccharomyces cerevisiae* yeast cells. Similarly, Margarita et al. (2016) found that the presence of *M. hominis* affects significantly a key phenomenon in *T. vaginalis* pathogenesis: lysis of host erythrocytes (red blood cells – RBCs). *In vitro T. vaginalis* hemolysis is an experimental model which led to the description and quantification of one of the potential mechanisms used by the protist to lyse target cells: the functional formation of pores on target cells membranes (Fiori et al., 1993). The presence of symbiotically associated *M. hominis* upregulates *T. vaginalis* hemolytic activity *in vitro* (Margarita et al., 2016). *In vivo* hemolysis occurring during natural *T. vaginalis* infection may lead to sustained parasite growth and may hence be involved in the exacerbation of symptoms observed in female patients during menses (Lehker et al., 1990).

Investigations on *in vitro* lysis of host cells by *T. vaginalis* are not limited to epithelial cells and RBCs: Mercer and colleagues studied the influence of *M. hominis* on the interaction of *T. vaginalis* with primary immune cells (B- and T-Lymphocytes, monocytes) (Mercer et al., 2016). While the presence of *M. hominis* did not affect the extent of *T. vaginalis-*mediated B- and T-lymphocytes lysis, the microbial symbiosis induced a qualitative and quantitative change in the proinflammatory cytokine milieu produced by monocytes. The host inflammatory response contributes to a great extent to *T. vaginalis* pathogenesis: the main cytological change observed during trichomoniasis is a heavy leukocyte infiltration leading to the production of proinflammatory cytokines (Shaio et al., 1995; Simhan et al., 2007). A study by Fiori et al. (2013) highlighted the profound influence of symbiotically associated *M. hominis* to the *in vitro* response of a human macrophage cell line to *T. vaginalis*. The bacterial symbiont sharply upregulates in a synergistic fashion the secretion of the proinflammatory cytokines IL-8, IL-1β, TNF-α, as well as the activation of the transcription factor NF-kB, which orchestrates many aspects of the innate immune response to infections. *M. hominis* hence appears to potentially increase the inflammatory processes observed during *T. vaginalis* infection. Inflammation is thought to have a key role in increased risk of HIV acquisition, and of cervical and prostate cancer associated with trichomoniasis (further discussed thereafter) (Yap et al., 1995; McClelland et al., 2007; Sutcliffe et al., 2012). In a speculative fashion, it could be hypothesized that the upregulation of the host inflammatory response to *Trichomonas* induced by *M. hominis* might also impact on these important pathologies associated with trichomoniasis.

Years of research clarified that the relationships between the human host and each of *T. vaginalis* and *M. hominis* are complex and multifaceted, and the symbiosis between these two microorganisms adds layers of complexity and profoundly influence the host/pathogen interactions.

### *T. vaginalis/M. hominis* Symbiosis and Female Reproductive Health

The colonization of *T. vaginalis* and *M. hominis* to the vaginal tract leads to important sequelae on fertility of women and has been associated with an increased risk in adverse pregnancy outcomes.

Female infertility is a common public health concern worldwide and endocrine, vaginal, cervical, uterine, tubal, and pelvic-peritoneal factors play an important role on its development. The most common causes of infertility is tubal factor infertility (TFI) characterized by inflammation of the epithelial surface of the fallopian tubes (salpingitis) and subsequent pelvic-peritoneal adhesions, both of which are mostly caused by previous or persistent infections (Tsevat et al., 2017). *T. vaginalis* along with *M. hominis* may potentially play roles in tubal damage although data associating these pathogens with TFI are scarce. Some retrospective studies have found that *T. vaginalis* prevalence is significantly higher among infertile women as compared to healthy controls, with approximately a two-fold risk increase of tubal infertility (El-Shazly et al., 2001). Moreover, by virtue of its cellular motility, *T. vaginalis* has been shown to be able to ascend the upper urogenital tract, contributing to the development of endometritis and salpingitis (Mielczarek and Blaszkowska, 2016). A recent article has confirmed the association between asymptomatic endocervical bacteria colonization and TFI, with a significant association with *M. hominis* detection (Piscopo et al., 2020). The infection of *T. vaginalis* symbiotically associated with *M. hominis* may increase the risk of upper urogenital tract infection, since trichomonads may spread *Mycoplasma* throughout the upper genital tract, indirectly eliciting tubal damage and infertility. These observations represent a starting point for additional research necessary to strengthen the suggestion that *T. vaginalis* and *M. hominis*, alone and in symbiosis, can contribute to infertility.

While the research about the role of *T. vaginalis* and *M. hominis* in women infertility appears to be in its infancy, the association between these pathogens and adverse pregnancy outcomes has been extensively studied. *M. hominis* is frequently isolated from both placental membranes and amniotic fluid in women with preterm prelabor rupture of membranes, with a potential direct effect of virulence mechanisms in this microenvironment (Choi et al., 2012; Capoccia et al., 2013). In contrast, the likely role of *T. vaginalis* in adverse pregnancy outcomes is given by the host immune response induced by local inflammation caused by infection, since the protist is a non-invasive pathogen unable to invade fetal membranes during pregnancy. The production of proinflammatory cytokines following trichomonad infection can indirectly lead to severe pregnancy complications (Fichorova, 2009; Mielczarek and Blaszkowska, 2016; Dessì et al., 2019). In a recent article, a gene (*goiC*) associated with amniotic infections and preterm delivery has been identified in *M. hominis* isolated in a group of women with preterm labor (Allen-Daniels et al., 2015). Interestingly, we have demonstrated that 58% of *M. hominis* intracellularly-associated with *T. vaginalis* have the *goiC* gene, suggesting an additional potential risk factor for adverse pregnancy outcomes during trichomoniasis (Thi Trung Thu et al., 2018).

Although metronidazole treatment for trichomonad infections is recommended for women at any stage of pregnancy (Sherrard et al., 2014), some authors have reported the failure of therapy of subclinical trichomoniasis to prevent adverse pregnancy outcomes (Klebanoff et al., 2001). This paradoxical result may be caused by the intracellular localization of *M. hominis* in *T. vaginalis*: the administration of drugs selectively effective against the protist could induce a massive release of *Mycoplasma* cells from dead parasites with subsequent bacterial invasion of fetal membranes and amniotic fluid (Thi Trung Thu et al., 2018).

The intracellular localization of *M. hominis* is protective for bacteria and can suggest a mechanism for transmission of infection from protists to human cervical and prostatic cells: as described in the case of amniotic fluid infections, anti-*T.vaginalis* treatment and/or cytolytic host immune response can kill protozoa allowing the massive delivering of compartmentalized *M.hominis* and subsequent bacterial invasion of host target cells.

The risk of complication in pregnant women affected by trichomoniasis treated with metronidazole is further supported by the fact that parasites treated with metronidazole release *Trichomonas vaginalis virus* (TVV) virions, a dsRNA virus frequently infecting trichomonad strains, which strongly activate the host-immune response, complicating the outcome in metronidazole-treated women and suggesting harmful sequelae during pregnancy (Fichorova et al., 2012).

### *T. vaginalis/M. hominis* Symbiosis and Cancer

An increasing number of microorganisms are considered to be involved in promotion, maintenance and progression of tumors (Gagnaire et al., 2017). This group includes mainly viruses and bacteria, but also some parasites are included in the list of pathogens able to induce tumors in humans, and it has been estimated that microbial infections contribute to about 1 in 4 of all tumors (Elinav et al., 2013; Porta et al., 2011). Cancer can be induced by direct mechanisms, following genetic and epigenetic alterations in host cells, or as a result of the synthesis of pro-oncogenic molecules by the pathogen; all these conditions can lead to development and progression of tumors (Bouvard et al., 2009). In addition, the induction of a local chronic inflammation in response to microbial infections, can represent a major cancer predisposition factor, exacerbating a tumor-supporting microenvironment. Interestingly, by analyzing a large number of tumoral tissues Nejman et al. (2020) demonstrated that different tumor types (e.g., bone, ovary, pancreas lung, breast, colon and melanoma) show distinct microbiome compositions, with a predominance of intracellular bacteria. However, it cannot be clearly established whether these microbial species could play a direct role in development and maintenance of cancer or are merely the result of secondary infections of tumoral tissues (Nejman et al., 2020).

Epidemiological, serological and experimental evidences suggest that *T.vaginalis* and its endosymbiont *M.hominis* may be both involved in direct and indirect mechanisms of oncogenesis. *T. vaginalis* infection has been considered as a risk factor for cervical cancer (Tao et al., 2014) as well as with aggressive prostate cancer (Sutcliffe et al., 2006) and prostate hyperplasia (Mitteregger et al., 2012). In addition, *Trichomonas* infection has been correlated with an increased risk of high-grade cervical cancer in patients with HPV16, suggesting that the protist may affect carcinogenicity of viruses (Yang et al., 2020).

In 2014 Twu and colleagues proposed a direct mechanism to explain the putative role of *T.vaginalis* in prostate cancer initiation and progression: the authors demonstrated that trichomonads are able to secrete a homologue of human Macrophage migration Inhibitory Factor (TvMIF), a well-known regulatory cytokine able to orchestrate proinflammatory responses, and biomarker of prostate cancer (Conroy et al., 2010). Beyond its characteristics as mediator of an inflammatory state, human MIF also seems to be involved in tumorigenesis due to its ability to inhibit p53 intracellular accumulation and subsequent cellular proliferation through activation of the MAPK family (Mitchell, 1999). TvMIF was experimentally shown to possess all the characteristics of its human ortholog, including the ability to bind the human specific receptor CD74 and to activate tyrosine kinases (Syk and PI3K/Akt) to activate the production of IL-6 and IL-8, and the ability to induce proliferation and invasiveness of normal and tumor-derived human prostate cells (Twu et al., 2014).

The infection by different *Mycoplasma* species may be related to the onset of tumors in humans, and the chronic nature of *Mycoplasma* infections suggests the establishment of a sub-clinical inflammation process potentially linked with pro-cancerous effects. Indeed, *Mycoplasma* infections have been associated with malignant transformation of mammalian cells (Feng et al., 1999; Namiki et al., 2009). Recently, *M. fermentans* has been associated with *in vivo* cellular transformation processes by influencing DNA-damage control and repair mechanisms via downregulation of p53 activity, thereby mimicking one the most common features of tumor cells (Zella et al., 2018).

More specifically, *M. hominis* infections have recently been associated with head and neck cancer (Atallah et al., 2020) and also to cervical tumors (Adebamowo et al., 2017; Klein et al., 2020). Furthermore Barykova and colleagues showed a significant link between *M. hominis* infections and the development of prostatic cancer (Barykova et al., 2011). Despite the relatively small size of the patient populations involved in the study, it could be speculated on the possible involvement of *M. hominis* in prostate cancer development, given that the bacterium was isolated at three times higher frequency in patients with prostate cancer than in those with benign prostatic hyperplasia, and in no case it was isolated from patients with no prostatic disease. Interestingly, some authors (Khan et al., 2016; Zakariah et al., 2018) proposed molecular mechanisms of carcinogenesis and identified a number of *M. hominis* proteins that are predicted to target regulatory processes involved in host cell cycle and apoptosis. It is thus tempting to hypothesize that chronic infections by *M. hominis* might be related to initiation, progression, and maintenance of cancer through induction of genomic instability and chromosomal aberrations in infected cells, probably due to its intracellular localization, inducing mitogenic and anti-apoptotic effect, and ultimately leading to malignant transformation.

The ability of *M. hominis* and *T. vaginalis* to establish a close symbiotic relationship opens new investigation hypotheses on the role of the pathogen consortium in the induction and maintenance of cancer. As previously discussed, the presence of *M. hominis* dramatically upregulates the host inflammatory response to *T. vaginalis*: since a marked chronic inflammatory state is a condition that predisposes the genital microenvironment to tumor transformation and maintenance, a role for the symbiotic relationship in regulating the oncogenic potential of the individual symbionts is a possibility deserving more attention by researchers.

Altogether, these studies suggest a hypothetical role in local tumor transformation and/or in the maintenance of a tumor-supporting microenvironment for the *T. vaginalis/M. hominis* holobiont: through a combination of chronic inflammation and secretion of bioactive molecules, such as TvMIF, this peculiar symbiosis might play a role in increased risk of human prostate and cervical cancer.

### *T. vaginalis/M. hominis* Symbiosis: Mutualism, Amensalism, Commensalism? An Evolutionary View

The relationship between *T. vaginalis* and *M. hominis* is a source of growing interest in the scientific community also from an evolutionary perspective: indeed, it is yet unclear whether this symbiosis can be described as mutualism or commensalism. In the previous paragraphs of this review we described several advantages for *M. hominis* deriving from this symbiotic association (intracellular localization protecting the bacterium from immune response and antibacterial drugs), while the potential gains for *T. vaginalis* are still elusive. In some respect the interaction between the microorganisms might be more akin to commensalism, with the bacterial endosymbionts deriving benefits and providing no apparent Darwinian advantage to the host protist in return. In order to clarify this issue, Margarita et al. (2016) shed light on the interactions between the two organisms further, demonstrating an increase in growth rate and ATP production in *mycoplasma*-infected *T. vaginalis* as compared to *mycoplasma*-free controls. The authors also investigated arginine metabolism: notably the two microorganisms share a metabolic pathway, Arginine Dihydrolase (ADH). Symbiotically associated *T. vaginalis* and *M. hominis* showed a pronounced increase in arginine consumption, with a potential increased competition with macrophages for arginine, limiting nitric oxide production of the macrophages and presumably the potential to exert the associated oxidative burst used to kill pathogens.

Another evidence for a potential considerable advantage gained by *M. hominis* establishing a symbiosis with the trichomonad protist was highlighted by Fürnkranz et al. (2018), by testing antibiotic susceptibilities of different *M. hominis* strains before and after co-culture with *T. vaginalis*. An increase in *M. hominis* resistance to clindamycin, moxifloxacin, ciprofloxacin and gentamicin after co-culturing with *T. vaginalis* was observed, but the exact mechanism of resistance is undetermined, yet it is indicative of more complex survival strategy, perhaps due to mutations in key drug resistance genes or increased efflux activity due to improved ATP synthesis as previously discussed.

Unfortunately, experimental data allowing to draw conclusions on the biological interconnection between *T. vaginalis* and *M. hominis* are still missing, and further work aimed at elucidating the true nature of this symbiotic relationship is needed.

## *Trichomonas* and *Acanthamoeba*: Really Unique Examples?

In this review we have focused primarily on the interactions of the protists *Acanthamoeba* spp. and *T. vaginalis* with their bacterial symbionts and discussed how these interactions may impact human health. In this aspect, the relationships established by *Trichomonas* and *Acanthamoeba* with their bacterial counterparts are unique examples of symbiosis affecting human health. Moreover, these two protists were selected as examples of different lifestyles: obligate parasite vs free-living. However, potential similar interactions with bacterial species have also been observed in other pathogenic protists such as *Giardia* spp. Sporadic researches suggest that *Giardia* spp. cells may harbor bacterial endosymbionts. Ultrastructural studies of *Giardia* trophozoites lead to the description of intracytoplasmic bacterial and mycoplasma-like structures (Feely et al., 1988); it should be noted that this is a mere morphological analysis, and no bacterial species has been isolated from *Giardia*, and no investigation aimed at assessing whether this observation is transient or the result of a stable biological association has been undertaken. This was later confirmed by an *ex-vivo* electron microscopy study conducted on mouse intestinal biopsies showing bacterial endosymbionts localized at the periphery of *Giardia muris* trophozoites. Interestingly, only bacteria-harboring trophozoites were killed by neighboring Paneth cells, while bacteria-free *Giardia* survived the immune killing mechanisms. The authors suggest a role for these unidentified putative bacterial endosymbionts in altering the protist’s antigenicity, leading to an immune cell activation which may on the one hand have a protective role for the host, on the other hand might contribute to pathology by triggering local inflammation (El-Shewy and Eid, 2005). These two studies can represent a strong clue that *Giardia* spp. is engaged in one or even more symbiotic relationship with bacteria, and further research aimed at a functional demonstration and characterization of this symbiosis would represent a new perspective on *Giardia* pathobiology.

*Entamoeba histolytica* is a parasite causing amoebiasis in humans where it thrives in the colon feeding on resident bacteria as its main nutritional source. Given its lifestyle surrounded by many different bacterial species, it could be reasonably expected that this environment might favor the establishment of a symbiotic relationship between the protist and a bacterial endosymbiont. However, to our knowledge no bacterial endosymbiont has ever been described for *E. histolytica*, while several clues suggest an important role in pathogenesis for the interaction with intestinal bacterial species, albeit these interactions cannot be described as symbiotic. When incubated with *E. coli* for instance, *E. histolytica* has an improved tolerance to oxidative stress treatments gained by bacteria-derived metabolites or enzymes such as oxaloacetate, malate dehydrogenase and catalase (Shaulov et al., 2018; Guillén, 2019). Protist/bacteria interactions on an evolutionary timescale are also a significant factor; in *Leishmania* spp., several mannosyltransferase/phosphorylase proteins are predicted to have been acquired through HGT from gram-positive bacteria during evolution. These proteins aid in the ability of *Leishmania* spp. to colonize new host niches and are essential for thermotolerance and the infection of the mammalian host (Sernee et al., 2019). Genomic evidence has demonstrated that HGT is not uncommon between bacterial species and parasitic protists (Nixon et al., 2003; Sernee et al., 2019) and the role this can play in human infections emphasizes the need to understand these relationships, especially to assess whether HGT is the result of protist predation over bacteria, or one of the possible outcomes of a hypothetical symbiotic relationship (Sibbald et al., 2020). An active investigation on the possibility that protists other than *T.vaginalis* and *Acanthamoeba*, especially *Giardia* spp., *Leishmania* spp., and *E. histolytica*, may have bacterial symbionts should be taken into account by parasitologists and bacteriologists alike.

## Discussion and Perspectives

Until the advent of the age of *-omics*, microbial pathogenesis has been a field of investigation which focused almost exclusively on the interactions of single pathogens with its host, both *in vitro* and *in vivo*, rather than taking into account that the animal hosts interact in each moment with complex microbial communities, the microbiota, composed of a plethora of species (viruses, bacteria and archaea, protists, and fungi) which in turn interact among each other with a number of beneficial and/or detrimental outcomes for the host. Recently, to reflect these interactions, the term ‘pathobiome’ was coined to describe scenarios, in which the interaction between multiple microbial species influences the health status of the host (Bass et al., 2019). Historically, the interactions of *Acanthamoeba* species with endosymbiotic human bacterial pathogens have been well characterized and indeed the role of *Acanthamoeba* as environmental reservoir for bacterial pathogens such as *L. pneumophila*, as well as a “biological gymnasium” where bacteria could possibly “train” and evolve intracellular survival mechanisms, represented a unique approach in the study of microbial virulence, the investigation of endosymbiotic relationships and the important consequences of their molecular and cellular mechanisms in human pathology. In this respect, the symbiosis between *Acanthamoeba* spp. and bacterial pathogens represented the sole example of a symbiosis between protists and bacteria with consequences for human health, until the detection of viable and replicating *M. hominis* within *T. vaginalis*. In this article we reviewed in detail the impact on pathogenesis of these two paradigmatic and unique protists/bacteria symbiotic relationships.

We described and discussed many aspects related to these two symbioses, and a key common factor emerging for both examples is a profound influence over host response and virulence of protists and bacteria alike. However, many open questions still need to be addressed. Firstly, many studies have utilized *Acanthamoeba* as a model to study phagocytosis (Leoni Swart et al., 2018) and experimental bacterial infection of *Acanthamoeba* can provide a “training ground” for subsequent infection of human cells, with certain bacteria often becoming more pathogenic after a passage in *Acanthamoeba* culture. The dissection of the molecular pathways and strategies, by which bacteria can become amoeba-resistant and resist phagocytosis could be beneficial for treating pathogens capable of preventing macrophage killing. Furthermore, how *Acanthamoeba* is able to survive these bacterial intracellular infections and subsequent multiplication is also worthy of investigation for new potential treatments. From a clinical point of view, the direct impact of *Acanthamoeba/*endosymbionts co-pathogenicity on clinical outcome of AK is debated, with some studies indicating it intensifies the pathology while others find no evidence to support this. Do the co-infections affect the host immune response and contribute to clinical variability? One possibility could be the phenomenon of HGT between *Acanthamoeba* and bacteria and between bacteria and other endosymbionts: does HGT contribute to drug resistance or virulence?

A traditional research approach to study *Acanthamoeba*/bacteria symbiosis effect usually take into account the interaction with one bacterial pathogen. In this review article we discussed also the possibility that interaction of *Acanthamoeba* with multiple microorganisms can lead to different clinical outcomes. More attention to the interaction between multiple bacterial symbionts, pathogenic and non-pathogenic, is needed.

The *T. vaginalis/M. hominis* symbiosis has impact on a number of different health conditions, but still further understanding is required in several areas.

First of all, several questions related to the influence of the symbiosis over host immune response need to be addressed. *M. hominis*, at least *in vitro*, was shown to trigger an upregulated host inflammatory response. Is symbiotically associated *M. hominis* involved in immune-related potential consequences of trichomoniasis, such as increased risk of HIV acquisition, and prostate and cervical cancer?

Both *T. vaginalis* and *M. hominis* can establish chronic infections. This implies a multifaceted interaction with the host immunity, especially with innate immune response: potential mechanisms of immune evasion need to be explored, as well as a hypothetical effect of the symbiosis on such mechanisms. By way of illustration, the recent identification and functional characterization of the *M. hominis* surface nuclease MHO_0730, potentially involved in evasion strategies from NETs discussed previously, raise the question whether symbiotically associated *M. hominis* might lend this ability to *T. vaginalis*.

Moreover, *M. hominis* can influence to a significant extent several aspects of *T. vaginalis* pathogenetic effect: does the symbiosis contribute to the wide range of variability of clinical manifestations of trichomoniasis?

Interestingly, *M. hominis* may not be the sole bacteria establishing a symbiosis with *T. vaginalis*. A few years ago a metagenomic analysis of the vaginal microbiomes of a population of individuals affected by trichomoniasis vs a control population led to the identification of genomic sequences of a previously unknown and unclassified *Mollicute*. The reconstruction of the complete genome sequence allowed the description of a previously unknown *Mycoplasma*, albeit it is presently still non-culturable: *Candidatus M. girerdii.* Notably, *M. girerdii* DNA could be found only in *T. vaginalis*-positive vaginal samples (Fettweis et al., 2014). The presence of *M. girerdii* in trichomoniasis patients populations has been the subject of just a few contradictory studies (Ioannidis et al., 2017; Masha et al., 2018; Jarrett et al., 2019), and its specific role as a novel *T. vaginalis* endosymbiont and its potential effect over pathogenesis is still an unexplored field of research deserving more attention.

An aspect of paramount importance which emerged as a consequence of many findings regarding pathological manifestations in which protist/bacteria symbioses play a role, is that in clinical infection the whole picture of all interacting microbial species potentially involved ‘pathobiome’, should be taken into account both in trichomoniasis and *Acanthamoeba* infections. Indeed, host colonization by one protist host entails also the acquisition of bacterial endosymbionts possibly present. This approach may indeed support increasing diagnosis accuracy and treatment effectiveness, opening new perspectives of intervention and infection control.

Importantly, evolutionary implications of the symbiotic relationships with bacteria established by *Acanthamoeba* and *Trichomonas* should also be taken into consideration: could these strict biological association be at least partly explained by evolutionary advantages for the symbionts? Are these symbioses still young on an evolutionary timescale? Could we expect unforeseeable impacts of the endosymbioses on the evolution of the multiple microbial species involved?

## Author Contributions

FH designed the structure of the review article, critically reviewed the manuscript, and coordinated the activities of coauthors involved in the *Acanthamoeba* section. RM contributed to the *Acanthamoeba* section and designed the figures related to *Acanthamoeba*. MZ contributed to the *Acanthamoeba* and bacteria paragraphs. EG contributed to the paragraph focused on *Acanthamoeba keratitis* and associated bacteria. TB contributed to the review of the amoeba-resistant bacteria (phagocytosis resistance). PF conceived the review article and contributed the “Tv/Mh symbiosis and cancer” paragraph. VM contributed to the “Tv/Mh symbiosis and female reproductive health” paragraph. PR contributed to the “*Trichomonas vaginalis”* and “*Trichomonas vaginalis* and *Mycoplasma hominis*: the discovery of a symbiotic relationship” paragraphs. DD conceived and designed the review article, critically reviewed the manuscript, coordinated coauthors activities. All authors contributed to the article and approved the submitted version.

## Conflict of Interest

The authors declare that the research was conducted in the absence of any commercial or financial relationships that could be construed as a potential conflict of interest.
